# Effects of Bypass Fat on Buffalo Carcass Characteristics, Meat Nutrient Contents and Profitability

**DOI:** 10.3390/ani11113042

**Published:** 2021-10-24

**Authors:** Amirul Faiz Mohd Azmi, Fhaisol Mat Amin, Hafandi Ahmad, Norhariani Mohd Nor, Goh Yong Meng, Mohd Zamri Saad, Md Zuki Abu Bakar, Punimin Abdullah, Agung Irawan, Anuraga Jayanegara, Hasliza Abu Hassim

**Affiliations:** 1 Department of Veterinary Preclinical Sciences, Faculty of Veterinary Medicine, Universiti Putra Malaysia (UPM), Serdang 43400, Selangor, Malaysia; amirulfaizazmi@gmail.com (A.F.M.A.); fhaisol@gmail.com (F.M.A.); hafandi@upm.edu.my (H.A.); norhariani@upm.edu.my (N.M.N.); ymgoh@upm.edu.my (G.Y.M.); zuki@upm.edu.my (M.Z.A.B.); 2 Department of Veterinary Laboratory Diagnosis, Faculty of Veterinary Medicine, Universiti Putra Malaysia (UPM), Serdang 43400, Selangor, Malaysia; mzamri@upm.edu.my; 3 Faculty of Science and Natural Resources, Universiti Malaysia Sabah, Jalan UMS, Kota Kinabalu 88400, Sabah, Malaysia; puniminabdullah@ums.edu.my; 4 Vocational School, Universitas Sebelas Maret, Surakarta 57126, Indonesia; a.irawan@staff.uns.ac.id; 5 Department of Animal and Rangeland Sciences, Oregon State University, Corvallis, OR 97333, USA; 6 Animal Feed and Nutrition Modelling (AFENUE) Research Group, Department of Nutrition and Feed Technology, Faculty of Animal Science, IPB University, Bogor 16680, Indonesia; anuraga.jayanegara@gmail.com; 7 Laboratory of Sustainable Animal Production and Biodiversity, Institute of Tropical Agriculture and Food Security, Universiti Putra Malaysia (UPM), Serdang 43400, Selangor, Malaysia

**Keywords:** buffalo, carcass, costs, meat, supplementation

## Abstract

**Simple Summary:**

Bypass fat supplementation has been shown to influence the carcass and meat qualities of large ruminants, especially cattle. However, limited information is available on the influence of bypass fat on carcass characteristics and the meat proximate and fatty acid compositions of buffaloes. The objective of this study was to evaluate both the effects of bypass fat on carcass traits and meat proximate and fatty acid compositions, and the profitability of Murrah cross and swamp buffaloes. Bypass fat supplementation improved the proximate and fatty acid compositions of buffalo meat without affecting the carcass characteristics. Although the mixture of the concentrate and bypass fat supplement (26:4) used in this study was found to increase the feed cost, the eventual overall returns resulted in a greater profit.

**Abstract:**

The deposition and distribution of buffalo body fats play a vital role in the quality of the buffalo carcass and are of great commercial value, since the carcass quality influences the profitability and consumer acceptability of ruminant meat. The current study examined the effect a mixture of 4% bypass fat and 26% concentrate supplementations in buffalo basal diet had on both the carcass characteristics and the proximate and fatty acid composition in *longissimus thoracis et lumborum* (LTL), *supraspinatus* (SS) and *semitendinosus* (ST) muscles of Murrah cross and swamp buffaloes. In addition, profit and loss analyses were performed to determine the profitability. This study employed a completely randomized 2 × 2 factorial arrangement with two diets, two breeds and four replicates per treatment. A total of sixteen buffaloes (eight buffaloes per breed, bodyweight 98.64 ± 1.93 kg) were randomly assigned into two dietary groups. The first group was given Diet A, which consisted of 70% *Brachiaria decumbens* + 30% concentrate, whereas the second group was given Diet B, which consisted of 70% *Brachiaria decumbens* + 26% concentrate + 4% bypass fat. The buffaloes were fed for 730 days before slaughter. The results showed that supplemented bypass fat significantly (*p* < 0.05) increased the pre-slaughter weight, hot and cold carcass weights, meat:fat ratio, pH at 24 h, moisture and crude protein of LTL, ST and SS, the ether extract of LTL and ST and the meat fatty acid of C16:0, C16:1, C18:1, PUFA n-6/n-3 and total MUFA. The carcass yield and carcass fat percentages, the ash content in ST, the EE in the SS muscle and the meat fatty acid of C18:3, total PUFA n-3, UFA/SFA and PUFA/SFA were significantly (*p* < 0.05) decreased. Furthermore, Murrah cross showed a significantly (*p* < 0.05) higher pre-slaughter weight, hot and cold carcass weights, carcass bone percentage and total fatty acid, but a lower (*p* < 0.05) meat:bone ratio, ash of LTL and CP of LTL and ST when compared to swamp buffaloes. No significant changes were found in the proximate composition of different types of muscle, but the ST muscle revealed significantly high C14:0, C16:0 and C18:1, and the SS muscle had high C18:2 and total fatty acid (*p* < 0.05). Supplementing using bypass fat increased the cost of buffalo feeding but resulted in a higher revenue and net profit. In conclusion, the concentrate and bypass fat supplementations in the buffalo diet could alter the nutrient compositions of buffalo meat without a detrimental effect on carcass characteristics, leading to a higher profit.

## 1. Introduction

Traditionally, buffalo meat was obtained from retired draft animals of more than 10 years of age [1]. Therefore, public perception remains that buffalo meats are tough and of low quality. However, when slaughtered at body weights equivalent to those of cattle, the carcass compositions and meat quality are comparable [2,3]. In fact, buffalo meat has a better tenderness compared to beef due to its high calpain activity in early post-mortem [4]. Therefore, buffalo meat has gained interest in recent decades due to its favored nutrient characteristics, such as a lower cholesterol and high protein contents [5].

Several factors contribute to the carcass quality of ruminants, including the feeding regimen, breed of animals and farm management [6]. Feed supplementation using commercialize concentrate and/or rumen bypass fat products from the local industry provide an inexpensive alternative to fulfil the nutritional demands of buffaloes [7,8,9]. Furthermore, enriching buffalo meat with good fatty acid through dietary supplementation allows buffalo meat to be more acceptable, particularly by health-conscious consumers. In fact, the fatty acid compositions of buffalo fat has been reported to affect the nutritional value and the various aspects of buffalo meat quality traits, including flavor and shelf-life [10], and the compositions of meat fatty acids [11]. An improvement in the ratio of polyunsaturated:saturated fatty acids (PUFA:SFA) in favor of the former is essential in maintaining the consumers’ health [12]. Fat supplements, such as palm, coconut and corn oils, given together with pasture, was shown to increase the percentage of fat in the carcass, thickness of meat-covering fat and marbling of the meat [7].

Crossbreeding of pure Murrah and swamp breeds is a common practice developed by buffalo farmers in Malaysia and other Asian countries, as the crossbreeding buffaloes were reported to inherit superior traits (e.g., growth, meat and milk production) possessed by their parents [9,13,14]. Indeed, the study by Mohd Azmi et al. [13] showed that the crossbreeds had a greater growth performance with a significantly heavier body weight than swamp buffaloes from birth until 24 months old. Other than nutritional factors, heterosis might have an impact on the growth and performance of buffaloes, which reflects the total production and profit of a farm [13,15].

Despite these superior traits and the potential use of crossbreeding Murrah and swamp buffaloes for meat purpose, it has received little attention. Indeed, the effects of bypass fat supplementation on carcass and meat quality traits in Murrah cross and swamp buffaloes are also scanty. Therefore, given the increasing importance of buffalo farming in developing countries, this study aimed to evaluate the impact of the dietary bypass fat supplements on the carcass quality and meat nutrient composition of Murrah crossbred and swamp buffaloes, as well as the profit and loss analysis.

## 2. Materials and Methods

### 2.1. Statement of Animal Rights

This study was performed and managed according to the Animal Utilization Protocol (AUP), Institutional Animal Care and Use Committee (IACUC) and Universiti Putra Malaysia (Approval No. UPM/IACUC/AUP-017/2018, on 8 January 2018). Samplings from the experimental animals were strictly conducted under veterinary supervision.

### 2.2. Study Area and Experimental Animals

This study was conducted at the Buffalo Breeding and Research Centre, Sabah, Malaysia (Coordinate 5°30′ N, 117°7′ E). A total of sixteen buffaloes, consisting of swamp (*n* = 8) and Murrah cross (*n* = 8) buffaloes of approximately 3 months old and with an average body weight of 98.64 ± 1.93 kg, were each randomly divided into two groups of 4 animals per group. The buffaloes were individually confined in collective stalls with an area of 30 m^2^ per animal, separated according to the dietary treatments. The feedlot facility had a compacted dirt floor, whereas the area close to the feeder was covered with concrete. The feeders were vinyl type and were placed transversely on the upper part of the pens, whereas the drinkers were located at the divider between two pens. Before the onset of the experiment, a proper physical examination was conducted on each buffalo. Then, all buffaloes were weighed and treated against ecto and endoparasites.

### 2.3. Experimental Design

This study was a completely randomized block design according to 2 × 2 factorial arrangement with two diets, two breeds and four replicates per treatment. Daily feed supply was calculated at 3% body weight (based on dry matter (DM) of total mixed ration), given in two equal portions at 07:00 h and 17:00 h [16]. Diets were adjusted to ensure that refusals were around 5% of the total supplied. The buffaloes were allowed a 14-day adaptation period to the respective diet before the start of the experiment. Two total mixed rations (TMR) were prepared to contain three ingredients; namely, *Brachiaria decumbens* grass (G), commercial concentrate (C) (composition: corn grain (25.0%), palm kernel cake (32.0%), rice bran (18.0%), soya bean meal (19.7%), calcium carbonate (1.0%), molasses (2.8%), vitamin–mineral premix (0.3%), sodium chloride (0.6%), dicalcium phosphate (0.6%)) and bypass fat (B) (OPTI-FAT F8016RXP—rumen bypass supplement sources from calcium salt fractionated palm fat without trans-fat). All diets were formulated similar to the previous study by Mohd Azmi et al. [13] in order to meet the nutritional requirements of growing buffalo [17,18,19]. The fresh *Brachiaria decumbens* grass (basal diet) was collected from the pastureland area of the farm at Telupid Buffalo Breeding and Research Centre, Sabah, Malaysia. The concentrate and bypass fat supplements were obtained from the authorized supplier, Lipidchem Sdn Bhd, Masai, Johor, Malaysia. Buffaloes of Group A were fed with Diet A, which consisted of 70% *Brachiaria decumbens* grass and 30% concentrate (Control) (DM: 90.31%, ash: 5.69% DM, crude fiber (CF): 23.73% DM, ether extract (EE): 2.92% DM, crude protein (CP): 8.08% DM, neutral detergent fiber (NDF): 57.96% DM, acid detergent fiber (ADF): 28.70% DM, acid detergent lignin (ADL): 3.32% DM, non-fiber carbohydrate (NFC): 24.84% DM, gross energy (GE): 12.10 MJ/kg, hemicellulose: 29.25% DM and cellulose: 25.38% DM), which is the common practice of buffalo feeding by farmers [13]. Group B was fed with Diet B, which consisted of 70% *Brachiaria decumbens* grass supplemented with 26% concentrate and 4% bypass fat (Treatment) (DM: 91.60%, ash: 5.93% DM, CF: 21.65% DM, EE: 6.66% DM, CP: 6.56% DM, NDF: 49.63% DM, ADF: 26.65% DM, ADL: 2.96% DM, NFC: 20.81% DM, GE: 14.59 MJ/kg, hemicellulose: 22.98% DM and cellulose: 23.69% DM) [13]. The fatty acid (FA) compositions of the feedstuffs and diets are presented in Table 1 and Table 2, respectively. The trial lasted 2 years before the buffaloes proceeded to slaughtering process.

### 2.4. Slaughtering Procedure and Sample Collection

Prior to slaughter, the buffaloes were placed at lairage for 12 to 16 h, but had access to drinking water. The buffaloes were weighed and slaughtered at a commercial government abattoir (Sabah Meat Technology Centre) according to the standard procedure (Muslim law; MS15000:2009) of the Department of Standard, Malaysia. After slaughtering, the weight of the warm carcass was immediately recorded before the head, fore and hind limbs were removed by cutting the atlantooccipital, carpal and tarsal joints, respectively [20]. Then, all carcasses were conditioned at 4 °C for 24 h before they were split longitudinally into two halves from the neck to pelvis along the vertebral column using a carcass splitting saw (Jarvis, CT, USA). Each primal cut was then physically dissected into lean, bone, fat and trimmable tissues, which consisted of major blood vessels, tendons, thick connective tissue sheets and glands [21].

Approximately 40 g of sample of *longissimus* *thoracis et lumborum* (LTL), *supraspinatus* (SS) and *semitendinosus* (ST) muscles were taken for the analyses. The samples were kept on ice during sampling, immediately vacuum-packed and stored at −20 °C until further analyses [22].

### 2.5. Carcass Traits

The parameters of carcass characteristics that were measured included the pH, the weight of the warm carcass, the weight of the cold carcass, the lean meat, the fat, the bone, the offal, the carcass yield and the shrinkage percentages. The pH of each carcass was measured using a portable pH meter (HANNA Hi8314 with an INGOLD type electrode Metrohm, Herisau, Switzerland) involved the left *longissimus* muscle, caudal to the 12th rib at 30 min, 24 h and 7 days post-mortem. Carcass yield was estimated using carcass weight over live weight and was expressed as a percentage [23]. The warm carcass weight was measured when dressed carcasses were quickly weighed within 1 h post-mortem, and the cold carcass weight was the weight of chilled carcass, which was kept at 4 °C for 24 h. Shrinkage percentage was calculated as the difference between warm and cold carcass weights and was expressed as a percentage [20]. The physical carcass composition was referred to as the ratio of major body tissues, muscle, fat and bone. The weights of these tissues were determined separately upon carcass dissection. The relationship between the weight of each carcass components and the carcass weight was computed and expressed as a percentage of the cold carcass weight.

### 2.6. Proximate Composition

The proximate compositions of feed samples and different type of muscles included the moisture, the ash, the ether extract, the crude protein and the carbohydrates. They were determined according to the method of the Association of Official Analytical Chemist [24], whereas the gross energy was determined using bomb calorimeter and expressed as MJ/kg sample.

### 2.7. Fatty Acid (FA) Analysis

The total fatty acids in the feed samples and the different type of muscles were extracted in chloroform:methanol (2:1, *v/v*) mixture according to the method of Folch et al. [25], as modified by Rajion et al. [26]. The fatty acids were transmethylated into their fatty acid methyl esters (FAME) using 0.66 N KOH in methanol and 14% methanolic boron trifluoride (BF3) following the method of AOAC [24]. The FAME was separated in a gas chromatograph (Agilent 7890A) equipped with a flame ionization detector (FID) and a split-less injector. The column used was fused silica capillary (Supelco SP-2560, 100 m, 0.25 mm ID, 0.20 mm film thickness). High purity helium was used as the carrier gas at 40 mL/min. Compressed air and high purity hydrogen were used for the FID in the chromatograph. To facilitate the optimal separation, the oven temperature was set at 100 °C for 2 min and warmed to 170 °C at 10 °C/min, held for 2 min, warmed to 230 °C at intervals of 5 °C/min and then held for 20 min. Identification of sample fatty acids was carried out by comparing the relative retention time of FAME peaks from samples with those of standards. Identification of fatty acids was carried out by comparing with the relative FAME peak retention times of fatty acid methyl standards using henecosanoic acid (Sigma, St. Louis, MO, USA) as the internal standard. The fatty acid was expressed as percentage from total fatty acid in each sample.

### 2.8. Profit and Loss Analysis of Buffalo Production

The economic aspect of buffalo meat production was calculated on the hot carcass weight at an exchange rate of USD 1.00 = MYR 4.07. To calculate the total operational cost, it was assumed that the cost of feeding represented 87% of the total cost of the activity [27], and the cost of feeding comprised the costs of basal diet and supplementations, which were concentrate and bypass fat [28]. At the time of the study, the values of the feed stuffs (MYR/kg) were MYR 0.23 (USD 0.06) for *Brachiaria decumbens*, MYR 1.11 (USD 0.27) for concentrate and MYR 3.82 (USD 0.94) for bypass fat. Previous study by Mohd Azmi et al. [13] reported that the average feed intake of Murrah cross buffaloes fed with Diet A was 6.57 kg/day and, for Diet B, it was 7.41 kg/day, whereas, for swamp buffaloes fed with Diet A, it was 6.15 kg/day and, for Diet B, it was 6.37 kg/day. The information of feed intake was used to calculate the total cost of average daily dry matter intake (MYR/day/animal). The 2-year management cost of MYR 158.50 (USD 38.94) per animal was added, which included the MYR 0.50 (USD 0.12) cost of deworming, MYR 2.00 (USD 0.49) for ID tag, MYR 156.00 (USD 38.33) for fertilizer, MYR 83.33 (USD 20.48) for transportation and MYR 152.00 (USD 37.35) for labor cost. Additional fixed cost imposed by the abattoir for slaughter services was priced at MYR 5.00/animal (USD 1.23/animal). The average price of fresh meat buffalo in Malaysia was MYR 31.03/kg (USD 7.62/kg), whereas the average price of bones was MYR 26.00/kg (USD 6.39/kg). The average price of head per animal was MYR 70.00 (USD 17.20/animal) and the average price of skin per animal was MYR 65.00 (USD 15.97/animal). The average price of tail per animal was MYR 10.00 (USD 2.46/animal) and butcher imposed overall offal (heart, liver, lung, limp, renal, intestine and rumen) price with value of MYR 35.00/kg (USD 8.60/kg). The gross profit of selling meat without the cost of aggregators (person who collects the animals from buffalo farm and transports them to livestock market for sale to sub traders) and traders or sub traders (registered person who supplies buffaloes to abattoirs, which incurs fees, such as transport cost, abattoir fees, miscellaneous expenditure and payments to sub-traders) was then calculated [29]. The income through meat price, the cost of feed per day for the 2-year rearing and the estimation of net income after selling fresh meat, bone and offal was calculated as below [28,30,31].

Meat sales (MYR/kg) = Meat weight (kg) × current price of fresh meat (MYR)

Bone sales (MYR/kg) = Bone weight (kg) × current price of bone (MYR)

Offal sales (MYR/kg) = Offal weight (kg) × current price of offal (MYR)

Total cost of average daily DMI (MYR/day/animal) = Current price of feed × dry matter intake (based on 3% body weight)

Total feed cost in 2 years (MYR/animal) = Total cost of average daily DMI × 720 days

### 2.9. Statistical Analysis

The data for the carcass characteristics and the profit and loss analyses were analyzed using analysis of variance (ANOVA) following a completely randomized block design in a 2 × 2 factorial arrangement (diet × breed) using MIXED command in SPSS. For these variables, dietary treatments and breeds were set as fixed effects and animal units within the breeds were declared as random effect. In addition, to test the differences in fatty acid profiles among types of muscles, one way ANOVA was performed. The data were presented as means and standard errors of the means. Tukey’s test was employed to test the differences between the means of the treatments and the differences were declared significant at *p* < 0.05 [32]. In addition, data of proximate composition of buffaloes’ muscle and fatty acid composition of the meat under different dietary treatments and breeds were compared using independent *t*-test.

## 3. Results

### 3.1. Carcass Traits

Table 3 shows the slaughter data of buffaloes that were fed with different diets. Significant (*p* < 0.05) interactions between the diet and breed were observed in the live weight and weights of the hot and cold carcass of the buffaloes. Buffaloes fed with Diet B were significantly higher in the live weight, the weights of the hot and cold carcass, the meat:fat ratio and the pH at 24 h, and were lower in the carcass fat percentage when compared to those fed with Diet A (*p* < 0.05). However, the carcass meat percentage was higher in the swamp buffalo fed with Diet B than that of Diet A (*p* < 0.05), whereas no such change was recorded in the Murrah cross. Furthermore, the breed significantly (*p* < 0.05) influenced the carcass quality, including the hot and cold carcass weights, the carcass bone percentage and the meat:bone ratio. The Murrah cross buffaloes were significantly (*p* < 0.05) higher in the live weight, the hot and cold carcass weights and the carcass bone percentage, but were lower in the meat:bone ratio than the swamp buffaloes. Moreover, there was a significant (*p* < 0.05) interaction between the diet and breed on the live weight (0.035), the hot carcass weight (0.047) and the cold carcass weight traits (0.041).

### 3.2. Proximate Composition of Different Types of Muscle

The effects of dietary treatments on the proximate composition of the buffalo LTL, ST and SS muscles are shown in Table 4. The inclusion of bypass fat in Diet B significantly (*p* < 0.05) increased the moisture, EE and CP contents of the LTL and SS, but lowered the ash content of ST. In fact, the increment of moisture content of ST was recorded only in swamp buffaloes fed with Diet B (*p* < 0.05). Furthermore, the breed significantly (*p* < 0.05) affected the ash and the CP of different muscles, with Murrah cross having a significantly (*p* < 0.05) lower ash content in the LTL and the CP of the LTL and the ST than the swamp buffaloes.

### 3.3. Fatty Acid Composition of Meat

The effects of different dietary treatments on the fatty acid composition of buffaloes’ muscle are shown in Table 5. The diet was found to significantly (*p* < 0.05) influence the fatty acid composition of meat. This was because buffaloes fed with Diet B had a higher (*p* < 0.05) C16:0, C16:1, C18:1, n6/n3 ratio and MUFA composition in the meat. On the other hand, the buffaloes fed with Diet A showed an increase in the meat fatty acid of C18:3, PUFA n-3, UFA/SFA ratio and PUFA/SFA ratio. This study also revealed that the buffaloes fed with Diet A had an increased ALA composition in the meat at 1.88-fold compared to the buffaloes fed with Diet B.

The fatty acid composition of the meat from different breeds of buffaloes are shown in Table 6. The Murrah cross buffaloes were significantly (*p* < 0.05) higher in linoleic acid (C18:2) and the total fatty acid composition than the swamp buffaloes (25.18% vs. 13.19% and 811.75 µg/mg vs. 610.48 µg/mg, respectively). The most abundant fatty acid in the buffaloes’ meats was alpha linoleic acid (C18:3) (45.39%), followed by linoleic acid (C18:2) (16.00%), oleic acid (C18:1) (8.54%), palmitic acid (C16:0) (8.30%) and stearic acid (C18:0) (2.68%), which accounted for approximately 75% to 80% of the total identified fatty acids in both buffalo breeds.

The fatty acid compositions in different types of buffaloes’ muscles (LTL, ST and SS) are shown in Table 7. Total fatty acid and C18:2 compositions were significantly (*p* < 0.05) higher in SS, followed by LTL and ST. Meanwhile, the compositions of C14:0 and C16:0 were higher (*p* < 0.05) in ST than SS and LTL. In addition, the composition of C18:1 was higher (*p* < 0.05) in ST and LTL, and lower in the SS muscle.

### 3.4. Profit and Loss Analysis of Buffalo Production

The effects of different dietary supplements on the cost of slaughtering buffaloes are shown in Table 8. The total cost, which is a summation of variable and fixed costs, was 20.28% higher (*p* < 0.05) for buffaloes fed with Diet B compared to Diet A. However, buffaloes fed with the diet supplementation had higher carcass, meat and bone weights, resulting in a higher total revenue, which ranged between MYR 4707.80 (USD 1156.71) and MYR 6260.13 (USD 1538.12) for the Murrah cross and between MYR 3782.76 (USD 929.43) and MYR 4521.50 (USD 1110.93) for the swamp buffaloes (*p* < 0.05). In fact, the concentrate and bypass fat supplements increased the total revenue for Murrah cross and swamp buffaloes by 24.80% and 16.34%, respectively, compared to the solely concentrate supplementation. Therefore, the total net profit was significantly (*p* < 0.05) higher in Diet B than Diet A by approximately 26.05% for Murrah cross buffaloes and 15.48% for swamp buffaloes. In addition, the Murrah cross buffaloes showed a significantly (*p* < 0.05) higher net profit than the swamp buffaloes.

## 4. Discussion

### 4.1. Carcass Quality

In the current study, certain carcass quality characteristic of buffaloes had a significant interaction between the diet and breed of buffaloes. Supporting these results, previous studies also reported that the diet and breed interaction influenced the livestock performance, carcass and meat quality characteristics [10,33,34]. This study was comparable with our finding that showed that the diet and breed had an impact on the pre-slaughter weight, as well as on the hot and cold carcass weight. Thus, the breed and the type of diets demonstrated a crucial role in the improvement and the optimization of the productivity and carcass traits of these buffaloes.

This study revealed that all parameters for the buffalo carcass characteristics were improved following feed supplementations. In fact, there was a significant interaction between the carcass weights and the diet, as well as the breed of buffalo when a heavier pre-slaughter weight and hot and cold carcass weights were recorded among the Murrah cross buffaloes fed with Diet B. Similarly, Bakker et al. [35] showed that the hot and cold carcass yield of cattle were greatly influenced by the type of supplements and breeds, while Di Stasio and Brugiapaglia [36] reported the same finding in buffaloes. It seems that a high energy diet produces a better live weight at slaughter, carcass yield and weights of internal organs and body fat [36,37,38].

Mohd Azmi et al. [13] reported that a supplementation of bypass fat was able to improve the growth performance, such as the average feed intake, the body weight and the body condition score of Murrah cross and swamp buffaloes, which might further influence the carcass quality traits. Indeed, Jones [39] reported that the carcass yield improvement was mainly due to the gains in the weight of muscle and bone, and the decrease in fat deposition, thereby increasing the weight of the bone and muscle, and decreasing the fat weight. Similarly, calves fed on high concentrate tended to have more fat than grazing, indicating that the short period of concentrate feeding before slaughter increased the fat depots in finisher calves [40]. On the contrary, there was no difference in the carcass yield and the body chemical composition [41], whereas a higher carcass yield and fat percentage by adding fat to the diet of lambs was reported [42]. It was reported that a lower level of fat supplementation (<5%) did not affect the carcass yield and body composition [43], whereas the lipid constituted the largest component of the carcass gain when cull cows were re-alimented with a high energy density diet [44].

The carcass yield, meat percentage and carcass meat weight are indicators used to evaluate slaughter performance [45]. The carcass yield is closely related to nutrient levels, breeds, age and feeding management [45]. In this study, the carcass fat and the carcass yield of buffaloes were slightly higher when they were fed with Diet A, due to the high percentage of concentrate in this diet. Earlier, Lambertz et al. [10] reported a higher carcass fat percentage in buffaloes following supplementation with a 2.0% body weight of concentrate, while Pimpa et al. [37] failed to improve the warm and cold carcass yield of the cattle when fed with 5% fat. Nevertheless, the average carcass yield of the Murrah cross and the swamp buffaloes in this study was markedly lower than the pure-breed Murrah buffaloes, as reported by Biswas and Rajkumar [3] and Arshadullah et al. [46], likely due to breed differences and the higher proportion of non-edible parts [16,47]. In fact, the Murrah cross in this study recorded a high carcass bone percentage (*p* < 0.05). Similar studies reported that Mediterranean pure Murrah, swamp and Murrah cross produce a high bone percentage, with an average of 25.63%, 18.50% and 16.37%, respectively [7,10,45]. The percentage of buffalo carcass bone, however, is not influenced by the diet, according to Bakker et al. [35].

The meat percentage is one of the most significant yields in carcass composition. It is influenced by the high-energy dietary energy intake [36]. Indeed, this study showed a significant increase in the carcass meat percentage and meat:fat ratio with Diet B. This was due to the adequate and continuous supply of a high energy density from the bypass fat and optimum protein provided by Diet B for growth and tissue maintenance [13,48], whereas the high concentrate in Diet A usually produces more carcass fat [48].

The pH of the post-slaughter meat is one of the factors that determines the quality of the carcass and meat [10,49]. Following the slaughter, the pH of the meat starts to decrease. In this study, the meat pH of Murrah cross fed with Diet A was significantly lower at 24 h than Diet B, but the pH of the meat of swamp buffaloes remained similar. Nevertheless, the range of the meat pH of this study was similar to the findings of Calabrò et al. [50] and Gecgel et al. [51].

### 4.2. Proximate Composition

It is well known that the nutrient properties of meat influence the meat quality, particularly the crude fat and crude protein contents and the fatty acid composition [52]. In this study, meat nutrient contents were found to be generally influenced by the diet and breed. The buffaloes fed with Diet B tended to have meat moisture, protein and fat contents that were significantly higher than the buffaloes fed with Diet A, probably due to the fact that feeding diets with a readily fermentable carbohydrate (concentrate) with bypass fat increased propionate production through ruminal fermentation and increased the insulin concentrations, which increased the intramuscular fat and protein syntheses [53,54]. Furthermore, the average protein content in buffalo meat was recorded as between 17.33% and 23.30%, and was significantly affected by the diets, and, to some extent, by the breed [55]. Our study revealed that the swamp buffaloes had a higher CP value in LTL and ST than the Murrah cross. The higher CP and fat contents in LTL than those of other topside meats [10] were probably due to the high myosin content, thus making the LTL juicier than other topsides [56]. Furthermore, since LTL is a less active muscle, it contains less collagen; thus, it is much softer, with a higher fat content than other topsides [57,58]. This study revealed that the swamp buffaloes had juicer meat than the Murrah cross. Similarly, the meat of the swamp buffaloes recorded a higher ash content in LTL than the Murrah cross (*p* < 0.05). Considering the swamp buffaloes as a draft animal, they probably store more iron in order to ensure there is enough myoglobin for their heavy workloads [9,59].

### 4.3. Fatty Acid Composition

In general, fatty acid compositions vary in the meat of water and river buffaloes. The most common fatty acid found in this study was C18:3, followed by C18:2, C18:1, C16:0 and C18:0, which were comparable with the results of Rao and Kowale [60], who reported a high C18:0, C18:1 and C18:2 composition in buffalo meat. However, Gecgel et al. [51] concluded that water buffaloes have a high C18:1 fatty acid composition, followed by C18:0 and C16:0. Meanwhile, river buffaloes were shown to be high in the fatty acid composition of C18:1, C16:1, C18:3 n-3 and C20:4 n-6 [10]. In this study, the percentages of the fatty acid composition for both breeds were comparable, but the Murrah cross recorded a significantly higher C18:2 and total fatty acid than the swamp buffaloes. A variation in the fatty acid composition between breeds of lambs was also reported by Budimir et al. [61], but the Italian Merino and Soravissana lambs had a similar fatty acids composition [61].

The buffalo SS muscle had a significantly high total fatty acid, and the LTL and ST had a high C18:2 composition, while the ST had a significantly high C14:0, C16:0 and C18:1 composition, as reported by Wood et al. [62] and Tamburrano et al. [63]. Similarly, Calabro et al. [50] reported significant differences between three types of muscles: the *longisimus thoracis, semitendinosus and iliopsas plus psoas minor*. In this study, all muscles showed a significant difference in the fatty acids composition, regardless of the type of diet offered. The meat of buffaloes fed with Diet B had a higher percentage of C16:0 and C16:1 than that of Diet A. Similarly, a study showed that Murrah buffaloes fed with a palm-based supplement significantly increased palmitic acid (C16:0) in the LTL muscle [64]. Furthermore, C18:1 and MUFA compositions in buffalo muscle were three-fold higher when fed with Diet B, but there was no difference in the C18:1 and MUFA compositions of the LTL muscle of Droper sheep fed with bypass fat [65]. Another study also revealed that Murrah buffalo fed with 1% body weight of either a palm, coconut or corn-based oil supplement had high C18:1 and MUFA concentrations in the LTL muscle [64]. The results of our study were in agreement with da Silva Lima et al. [66] and Andrade et al. [67], who studied in cattle. They reported that cattle showed a higher proportion of UFA, namely C18:1, followed by SFA, such as C16:0. Therefore, the difference in the C18:1 composition in the muscle might be due to the different proportions of diet given to the animals. In addition, the biohydrogenation process within the rumen contributed a small proportion of fatty acid [68,69]. Therefore, buffaloes that were fed Diet A had a lower content of MUFA compared to those on Diet B.

The presence of n-3 fatty acids helps to determine the buffalo meat’s nutrient quality [63]. The n-3 fatty acid, in particular, has the potential to reduce the serum concentration of triglycerides in humans [10]. In this study, n-3 fatty acid was present at a low level in the buffalo muscle fed with Diet B. The low level might be due to the low intensity of C18:3 n-3 ruminal biohydrogenation [70] in the rumen, which led to a low composition of C18:3 n-3 in the meat. Due to the low amount of C18:3 n-3, the subsequent level of total n-3 fatty acids was significantly low, leading to a significantly high n-6/n-3 ratio compared to Diet A (*p* < 0.05). Therefore, the ratio of n-6/n-3 fatty acid in the meat is influenced by the diet [51]. Even though there was a high n-6/n-3 ratio with Diet B, it is still considered to be a healthy meat [71], since it falls within the recommended ratio of the World Health [72].

Cifuni et al. [73] concluded that the fat mass percentage in the animal muscle could influence the fatty acid composition of both meat and PUFA/SFA and UFA/SFA fatty acid ratios. Buffaloes fed with Diet B in this study had a lower carcass fat percentage compared to Diet A (*p* < 0.05), and, subsequently, were low in UFA/SFA and PUFA/SFA ratios. Indeed, the PUFA/SFA ratio is recommended to be low, since it is considered to be beneficial for human health [63]. Nevertheless, the content of the fatty acid in this study was different from that reported by Gecgel et al. [51] and Lambertz et al. [10]. This might be due to the diet and breed [74], but Lima et al. [75] and Silva et al. [76] found little differences in the fatty acid composition of cattle meat that included protected fat in the diets. These differences were potentially due to differences in the animal study, type and ratio of feed offered and muscle functionality. Diets that contain high concentrations of fat were either partially or entirely protected from microbial action in the rumen, thus, causing changes in the fatty acid composition, which may lead to an increase in the intramuscular fat deposition [67,77].

### 4.4. Profit and Loss Analysis of Buffalo Production

In feedlot production, a farmer needs to sell the reared animal as soon as possible in order to ensure a consistent cash inflow. To reduce the costs of the animal meat production, farmers are increasingly seeking alternative feed additives, particularly cheaper feedstuffs with desired qualities that enable the animal to reach a market weight and slaughter weight within the given rearing period. An alternative that attracts a great deal of interest is the use of concentrate, either solely or in a mixture with bypass fat [10,66,78]. However, excessive amounts of dietary lipids might result in a negative effect on fiber digestion in the rumen and may influence the quality of the meat and the cost of animal feed [75], which makes the decision to change to the alternative difficult. With the right proportion of supplementations used in this study, the adverse effects on meat quality and costs could be controlled.

This study revealed that the 2-year operating expenses were significantly (*p* < 0.05) different between Murrah cross and swamp buffaloes. Diet B cost between MYR 2570.45 (USD 631.56) and MYR 2266.16 (USD 556.80), whereas Diet A cost between MYR 1979.27 (USD 486.31) and MYR 1876.57 (USD 461.07). In fact, additions of feed ingredients, such as concentrate or bypass fat, have been reported to increase the feed cost [79]. In this study, the net profit was significantly (*p* < 0.05) higher with Diet B than Diet A, at the rate of 26.05% for Murrah cross buffaloes and 15.48% for swamp buffaloes. However, a proper supplement ratio should be able to improve the growth performance and the carcass characteristics of the ruminant, thus becoming cost-effective with the potential of enhancing the profit [80,81,82]. Overall, diets with a supplement for the Murrah cross resulted in a higher net profit than that of swamp buffaloes. The result can improve the awareness on costs of production to farmers and policy makers and can enable them to make appropriate decisions when changing farm management. More research on the cost effectiveness of diets with a supplement on buffalo health should be performed in the future.

## 5. Conclusions

Supplementations of pasture with 26% concentrate and 4% bypass fat enhanced the carcass quality (i.e., hot and cold carcass weights and meat:fat ratio) and the proximate compositions of different buffaloes’ muscles (i.e., moisture and crude protein of LTL, ST and SS and the ether extract of LTL and ST), as well as the meat fatty acid (i.e., C16:0, C16:1, C18:1, PUFA n-6/n-3 and total MUFA). The Murrah cross showed a significantly better carcass quality (hot and cold carcass weights and carcass bone yield), with a higher C18:2 and total fatty acid content, whereas the proximate composition of meat (i.e., ash of LTL and crude protein of LTL and ST) was better in swamp buffaloes. The supplementation of bypass fat in the buffalo diet significantly increased the cost of feeding, but eventually resulted in a significantly higher revenue and net profit. Further studies should address the meat quality and the nuclear magnetic resonance metabolic profiles of Murrah cross and swamp buffaloes supplemented with concentrate and bypass fat in order to explore the compounds that contribute to the physiochemical properties, sensory assessment and nutritional quality of food.

## Figures and Tables

**Table 1:** Fatty acid compositions of feedstuffs.

	Feedstuffs		
Fatty Acids Composition (% Total FA) ^†^	Grass	Concentrate	Bypass Fat
C12:0 Lauric acid	0.75	6.70	0.51
C14:0 Myristic acid	0.3	1.96	0.16
C16:0 Palmitic acid	2.03	51.47	51.23
C18:0 Stearic acid	8.43	10.12	0.43
C18:1 n-9, Oleic acid	45.87	19.90	47.44
C18:2 n-6, Linoleic acid	40.44	8.10	0.10
C18:3 n-3, Alpha linoleic acid	1.93	1.37	0.10
C20:0 Eicosanoic acid	0.24	0.38	0.05
Total fatty acid (µg/mg)	192.35	556.40	2861.57
Σ SFA ^1^	11.75	70.63	52.39
Σ UFA ^2^	88.25	29.37	47.65
Σ PUFA n-3 ^3^	1.93	1.37	0.10
Σ PUFA n-6 ^4^	40.44	8.10	0.10
Σ PUFA ^5^	42.38	9.47	0.20
PUFA n-6/n-3 ratio	20.91	5.90	1.05
Σ MUFA ^6^	45.87	19.90	47.44

Note: ^1^ SFA (saturated fatty acids) = C12:0 + C14:0 + C16:0 + C18:0 + C + 20:0; ^2^ UFA (unsaturated fatty acids) = C18:1 + C18:2 + C18:3; ^3^ PUFA n-3 = C18:3 n-3; ^4^ PUFA n-6 = C18:2 n-6; ^5^ Total PUFA = PUFA n-3 + PUFA n-6; ^6^ MUFA (monounsaturated fatty acids) = C18:1. ^†^ The result of individual fatty acid was expressed as percentage from total fatty acid in each sample.

**Table 2:** Fatty acid compositions of total mixed ration.

	Diets	
Ingredient (%)	Diet A	Diet B
*Brachiaria decumbens* (G)	70	70
Concentrate (C)	30	26
Bypass fat (B)	-	4
Total	100	100
Fatty acids composition (% total FA) ^†^		
C12:0 Lauric acid	2.60	2.13
C14:0 Myristic acid	1.01	0.76
C16:0 Palmitic acid	11.02	59.60
C18:0 Stearic caid	55.90	18.83
C18:1 n-9, Oleic acid	13.26	9.58
C18:2 n-6, Linoleic acid	13.67	8.65
C18:3 n-3, Alpha linoleic acid	1.04	0.46
C20:0 Eicosanoic acid	0.22	0.04
Total fatty acid (µg/mg)	543.10	674.09
Σ SFA ^1^	70.75	81.35
Σ UFA ^2^	29.25	18.39
Σ PUFA n-3 ^3^	1.04	0.46
Σ PUFA n-6 ^4^	13.67	8.65
Σ PUFA ^5^	14.71	9.11
PUFA n-6/n-3 ratio	13.19	18.87
Σ MUFA ^6^	13.26	9.58

Note: ^1^ SFA (saturated fatty acids) = C12:0 + C14:0 + C16:0 + C18:0 + C + 20:0; ^2^ UFA (unsaturated fatty acids) = C18:1 + C18:2 + C18:3; ^3^ PUFA n-3 = C18:3 n-3; ^4^ PUFA n-6 = C18:2 n-6; ^5^ Total PUFA = PUFA n-3 + PUFA n-6; ^6^ MUFA (monounsaturated fatty acids) = C18:1. ^†^ The result of individual fatty acid was expressed as percentage from total fatty acid in each sample.

**Table 3:** Effect of supplementation and breed, and their impact on buffaloes’ carcass traits.

Breed	Murrah Cross		Swamp			*p*-Value		Interaction
Diets	Diet A ^1^	Diet B	Diet A	Diet B	SEM ^2^	Diet	Breed	Diet × Breed
Pre-slaughter weight (kg)	330.75 ^aY^	451.70 ^bY^	281.75 ^aZ^	353.78 ^bZ^	16.15	*	*	0.035
Hot carcass (kg)	157.08 ^aY^	204.10 ^bY^	131.33 ^aZ^	150.28 ^bZ^	7.23	*	*	0.047
Cold carcass (kg)	144.53 ^aY^	192.60 ^bY^	120.28 ^aZ^	139.40 ^bZ^	7.19	*	*	0.041
Carcass yield (%)	47.50 ^a^	45.17 ^b^	46.66 ^a^	42.41 ^b^	0.70	*	ns	ns
Shrinkage (%)	7.99	5.62	8.40	7.30	0.46	ns	ns	ns
Fat (%)	12.35 ^a^	6.49 ^b^	8.23 ^a^	5.54 ^b^	0.78	*	ns	ns
Bone (%)	21.85 ^Y^	23.85 ^Y^	19.02 ^Z^	18.12 ^Z^	1.01	ns	*	ns
Meat (%)	59.01 ^a^	59.20 ^b^	56.91 ^a^	60.75 ^b^	0.81	*	ns	ns
Meat:bone ratio	2.74 ^Y^	2.45 ^Y^	3.22 ^Z^	3.43 ^Z^	0.18	ns	*	ns
Meat:fat ratio	5.05 ^a^	9.43 ^b^	6.98 ^a^	11.33 ^b^	0.75	*	ns	ns
Offal (% BW)	6.87	6.92	6.79	6.81	0.16	ns	ns	ns
pH (0 h)	6.77	6.69	6.67	6.81	0.06	ns	ns	ns
pH (24 h)	5.51 ^a^	5.87 ^b^	5.43 ^a^	5.56 ^b^	0.06	*	ns	ns
pH (48 h)	5.48	5.38	5.43	5.35	0.03	ns	ns	ns

Note: ^1^ Diet A = 70% *Brachiaria decumbence* + 30% concentrate, Diet B = 70% *Brachiaria decumbence* + 26% concentrate + 4% bypass fat; ^2^ SEM = standard error of mean; ^a,b^ values with different superscripts indicate significant difference between dietary treatments at *p* < 0.05; ^Y,Z^ values with different superscripts indicate significant difference between breeds at *p* < 0.05; % = percentage based on the carcass weight; % BW = percentage based on pre-slaughter body weight; * = *p* < 0.05, ns = not significant.

**Table 4:** Proximate composition of different type of muscles in Murrah cross and swamp buffaloes under different dietary treatments.

Nutrient Composition of Different Type of Muscles	Breeds				Dietary Treatments			
	Murrah Cross	Swamp	SEM	*p*-Value	Diet A ^1^	Diet B	SEM ^2^	*p*-Value
*Longissimus thoracis et lumborum* (LTL)								
Moisture (%)	73.75	73.61	0.05	ns	71.37 ^b^	75.99 ^a^	1.63	*
Ash (%)	1.39 ^Z^	1.78 ^Y^	0.14	*	1.64	1.53	0.04	ns
Ether extract (%)	2.82	2.90	0.03	ns	2.13 ^b^	3.59 ^a^	0.52	*
Crude protein (%)	24.31 ^Z^	25.58 ^Y^	0.45	*	24.39 ^b^	25.49 ^a^	0.39	*
Carbohydrates (%)	nd	nd	-	ns	nd ^3^	Nd	-	ns
Gross energy (MJ/kg)	21.18	20.66	0.18	ns	20.35	21.48	0.40	ns
*Semitendinosus* (ST)								
Moisture (%)	70.27	73.08	1.00	ns	70.64 ^b^	72.71 ^a^	0.73	*
Ash (%)	1.29	1.28	0.01	ns	1.64 ^a^	0.93 ^b^	0.25	*
Ether extract (%)	3.04	2.00	0.37	ns	1.52 ^b^	3.52 ^a^	0.71	*
Crude protein (%)	22.62 ^Z^	26.57 ^Y^	1.40	*	23.42 ^b^	25.77 ^a^	0.83	*
Carbohydrates (%)	4.71	1.24	1.23	ns	2.98	nd ^3^	-	ns
Gross energy (MJ/kg)	21.27	20.96	0.11	ns	20.80	21.44	0.23	ns
*Supraspinatus* (SS)								
Moisture (%)	73.96	74.71	0.27	ns	72.53 ^b^	76.13 ^a^	1.27	*
Ash (%)	1.81	1.60	0.08	ns	1.67	1.74	0.02	ns
Ether extract (%)	2.96	2.15	0.29	ns	2.38 ^b^	2.73 ^a^	0.12	*
Crude protein (%)	26.38	24.38	0.71	ns	23.76 ^b^	27.00 ^a^	1.14	*
Carbohydrates (%)	0.72	1.83	0.39	ns	1.28	nd^3^	-	ns
Gross energy (MJ/kg)	21.19	21.02	0.06	ns	21.24	20.97	0.10	ns

Note: ^1^ Diet A (control): 70% *Brachiaria decumbence* + 30% concentrate, Diet B: 70% *Brachiaria decumbence* + 26% concentrate + 4% bypass fat; ^2^ SEM = standard error of mean; ^3^ nd = not determined; * = *p* < 0.05, ^a,b^ values with different superscripts indicate significant difference between dietary treatments at *p* < 0.05; ^Y,Z^ values with different superscripts indicate significant difference between breeds at *p* < 0.05; n.s = not significant.

**Table 5:** Fatty acid composition of buffalo meat under different dietary treatments.

Fatty Acid Composition (% Total FA) ^†^	Dietary Treatments		SEM ^9^	*p*-Value
	Diet A ^1^	Diet B		
C14:0 Myristic acid	0.56	2.33	0.63	ns
C15:0 Pentadecanoic acid	1.55	0.77	0.28	ns
C15:1 Pentadecanoic acid (cis-10)	1.29	2.36	0.38	ns
C16:0 Palmitic acid	3.36 ^B^	15.04 ^A^	4.13	*
C16:1 Palmitoleic acid	1.09 ^B^	1.87 ^A^	0.28	*
C17:0 Heptadecanoic acid	2.10	3.09	0.35	ns
C17:1 Heptadecenoic acid	2.47	2.07	0.14	ns
C18:0 Stearic acid	2.04	2.32	0.10	ns
C18:1 n9c, Oleic acid	4.01 ^B^	13.18 ^A^	3.24	*
C18:2 n6c, Linoleic acid	20.33	18.36	0.70	ns
C18:3 n3c, Alpha linoleic acid	53.85 ^A^	28.63 ^B^	8.92	*
C20:0 Eicosanoic acid	0.45	0.32	0.05	ns
C20:1 n9c, Eicosenoic acid	0.19	0.28	0.03	ns
C20:2 n6c, Eicosadienoic acid	1.20	0.62	0.21	ns
C20:3 n6c, Dihomo-γ-linoleic acid	2.55	4.22	0.59	ns
C20:4 n6c, Arachidonic acid	1.17	2.83	0.59	ns
C20:5 n3c, Eicosapentanoic acid	1.52	0.61	0.32	ns
C22:0 Docosanoic acid	0.11	0.17	0.02	ns
C22:1 n9c, Erucic acid	0.56	0.69	0.05	ns
C22:2 n6c, Docosadienoic acid	0.14	0.26	0.04	ns
C22:6 n3c, Docosahexanoic acid	0.38	0.25	0.05	ns
Total fatty acid (µg/mg)	766.75	727.90	13.74	ns
Total SFA ^2^	9.65	22.96	4.71	ns
Total UFA ^3^	81.10	72.48	3.05	ns
Total PUFA n-3 ^4^	55.66 ^A^	29.44 ^B^	9.27	*
Total PUFA n-6 ^5^	18.58	2 3.66	1.80	ns
Total PUFA n-9 ^6^	3.41	13.43	3.54	ns
Total PUFA ^7^	77.66	66.52	3.94	ns
PUFA n-6/n-3 ratio	0.35 ^B^	0.93 ^A^	0.21	*
UFA/SFA	10.19 ^A^	4.17 ^B^	2.13	*
PUFA/SFA	9.80 ^A^	3.91 ^B^	2.08	*
Total MUFA ^8^	6.16 ^B^	18.50 ^A^	4.36	*

Note: ^1^ Diet A (control): 70% *Brachiaria decumbence* + 30% concentrate, Diet B: 70% *Brachiaria decumbence* + 26% concentrate + 4% bypass fat; ^2^ Total SFA (saturated fatty acids) = C14:0 + C15:0 + C16:0 + C17:0 + C18:0 + C + 20:0 + C22:0; ^3^ Total UFA (unsaturated fatty acids) = C15:1 + C16:1 + C17:1 + C18:1 + C18:2 + C18:3 + C20:4 + C20:5 + C22:6; ^4^ Total PUFA n-3 = C18:3 n-3 + C20:5 n-3 + C22:6 n-3; ^5^ Total PUFA n-6 = C18:2 n-6 + C20:2 n-6 + C20:3 n-6 + C20:4 n-6 + C22:2 n-6; ^6^ Total PUFA n-9 = C18:1 n9 + C20:1 n9 + C22:1 n-9; ^7^ Total PUFA = PUFA n-3 + PUFA n-6 + PUFA n-9; ^8^ Total MUFA (monounsaturated fatty acids) = C15:1 + C16:1 + C17:1 + C18:1; ^9^ SEM = standard error of mean. ^†^ The result of individual fatty acid was expressed as percentage from total fatty acid in each sample; ^A,B^ values with different superscripts within a row differ significantly at *p* < 0.05; * indicates that the value is significantly different at *p* < 0.05; ns indicates that the value is not significantly different at *p* < 0.05.

**Table 6:** Fatty acid composition of meat from different breed of buffaloes.

Fatty Acid Composition (% Total FA) ^†^	Breed		SEM ^9^	*p*-Value
	MC ^1^	SW		
C14:0 Myristic acid	1.17	1.70	0.19	ns
C15:0 Pentadecanoic acid	0.74	1.36	0.22	ns
C15:1 Pentadecanoic acid (cis-10)	1.81	1.61	0.07	ns
C16:0 Palmitic acid	8.30	10.78	0.88	ns
C16:1 Palmitoleic acid	0.94	2.46	0.54	ns
C17:0 Heptadecanoic acid	1.85	3.54	0.60	ns
C17:1 Heptadecenoic acid	1.34	2.92	0.56	ns
C18:0 Stearic acid	3.04	2.63	0.14	ns
C18:1 n9c, Oleic acid	10.69	11.02	0.12	ns
C18:2 n6c, Linoleic acid	25.18 ^y^	13.19 ^z^	4.24	*
C18:3 n3c, Alpha linoleic acid	36.58	39.47	1.02	ns
C20:0 Eicosanoic caid	0.30	0.39	0.03	ns
C20:1 n9c, Eicosenoic acid	0.23	0.26	0.01	ns
C20:2 n6c, Eicosadienoic acid	0.72	1.24	0.18	ns
C20:3 n6c, Dihomo-γ-linoleic acid	2.95	3.78	0.29	ns
C20:4 n6c, Arachidonic acid	2.27	1.90	0.13	ns
C20:5 n3c, Eicosapentanoic acid	0.69	1.47	0.28	ns
C22:0 Docosanoic acid	0.15	0.16	0.00	ns
C22:1 n9c, Erucic acid	0.57	0.65	0.03	ns
C22:2 n6c, Docosadienoic acid	0.24	0.16	0.03	ns
C22:6 n3c, Docosahexanoic acid	0.42	0.22	0.07	ns
Total fatty acid (µg/mg)	811.75 ^y^	610.48 ^z^	71.16	*
Total SFA ^2^	14.29	20.40	2.16	ns
Total UFA ^3^	75.44	77.79	0.83	ns
Total PUFA n-3 ^4^	37.55	41.14	1.27	ns
Total PUFA n-6 ^5^	24.66	19.11	1.96	ns
Total PUFA n-9 ^6^	9.45	11.91	0.87	ns
Total PUFA ^7^	71.66	72.16	0.18	ns
PUFA n-6/n-3 ratio	0.81	0.67	0.05	ns
UFA/SFA	7.98	5.26	0.96	ns
PUFA/SFA	7.63	4.99	0.93	ns
Total MUFA ^8^	12.56	16.65	1.45	ns

Note: ^1^ MC = Murrah cross buffalo, SW = swamp buffalo; ^2^ Total SFA (saturated fatty acids) = C14:0 + C15:0 + C16:0 + C17:0 + C18:0 + C + 20:0 + C22:0; ^3^ Total UFA (unsaturated fatty acids) = C15:1 + C16:1 + C17:1 + C18:1 + C18:2 + C18:3 + C20:4 + C20:5 + C22:6; ^4^ Total PUFA n-3 = C18:3 n-3 + C20:5 n-3 + C22:6 n-3; ^5^ Total PUFA n-6 = C18:2 n-6 + C20:2 n-6 + C20:3 n-6 + C20:4 n-6 + C22:2 n-6; ^6^ Total PUFA n-9 = C18:1 n9 + C20:1 n9 + C22:1 n-9; ^7^ Total PUFA = PUFA n-3 + PUFA n-6 + PUFA n-9; ^8^ Total MUFA (monounsaturated fatty acids) = C15:1 + C16:1 + C17:1 + C18:1; ^9^ SEM = standard error of mean; ^†^ The result of individual fatty acid was expressed as percentage from total fatty acid in each sample; ^y,z^ values with different superscripts within a row differ significantly at *p* < 0.05; * indicates that the value is significantly different at *p* < 0.05; ns indicates that the value is not significantly different at *p* < 0.05.

**Table 7:** Fatty acid composition of different types of buffalo muscle.

Fatty Acid Composition (% Total FA) ^†^	Type of Muscle			SEM ^9^	*p*-Value
	LTL ^1^	ST	SS		
C14:0 Myristic acid	0.84 ^b^	2.55 ^a^	0.93 ^b^	0.47	*
C15:0 Pentadecanoic acid	1.38	0.75	1.03	0.08	ns
C15:1 Pentadecanoic acid (cis-10)	1.85	0.91	2.36	0.42	ns
C16:0 Palmitic acid	6.76 ^c^	12.80 ^a^	9.06 ^b^	1.08	*
C16:1 Palmitoleic acid	1.03	3.86	0.23	1.05	ns
C17:0 Heptadecanoic acid	2.69	2.93	2.47	0.13	ns
C17:1 Heptadecenoic acid	1.46	3.34	1.58	0.51	ns
C18:0 Stearic acid	4.91	2.14	1.45	0.20	ns
C18:1 n9c, Oleic acid	12.02 ^b^	14.26 ^a^	6.28 ^c^	2.30	*
C18:2 n6c, Linoleic acid	19.14 ^b^	10.54 ^c^	27.87 ^a^	5.00	*
C18:3 n3c, Alpha linoleic acid	40.17	36.08	37.82	0.50	ns
C20:0 Eicosanoic acid	0.43	0.34	0.28	0.02	ns
C20:1 n9c, Eicosenoic acid	0.22	0.28	0.23	0.01	ns
C20:2 n6c, Eicosadienoic acid	1.94	0.54	0.46	0.02	ns
C20:3 n6c, Dihomo- γ-linoleic acid	2.81	3.89	3.40	0.14	ns
C20:4 n6c, Arachidonic acid	1.66	1.86	2.74	0.25	ns
C20:5 n3c, Eicosapentanoic acid	1.17	1.32	0.75	0.16	ns
C22:0 Docosanoic acid	0.17	0.10	0.19	0.03	ns
C22:1 n9c, Erucic acid	0.44	1.06	0.32	0.21	ns
C22:2 n6c, Docosadienoic acid	0.19	0.16	0.25	0.03	ns
C22:6 n3c, Docosahexanoic acid	0.36	0.30	0.32	0.01	ns
Total fatty acid (µg/mg)	652.74 ^b^	628.02 ^b^	852.59 ^a^	64.83	*
Total SFA ^2^	17.00	20.92	14.11	1.97	ns
Total UFA ^3^	83.00	71.37	75.49	1.19	ns
Total PUFA n-3 ^4^	41.64	37.58	38.82	0.36	ns
Total PUFA n-6 ^5^	24.86	14.65	26.14	3.32	ns
Total PUFA n-9 ^6^	12.62	12.66	6.77	1.70	ns
Total PUFA ^7^	79.11	64.89	71.73	1.97	ns
PUFA n-6/n-3 ratio	0.88	0.60	0.74	0.04	ns
UFA/SFA	6.29	5.91	7.65	0.50	ns
PUFA/SFA	6.01	5.54	7.37	0.53	ns
Total MUFA ^8^	15.90	17.95	9.97	2.30	ns

Note: ^1^ LTL = *longissimus thoracis et lumborum*; ST = *semitendinosus*; SS = *supraspinatus*; ^2^ Total SFA (saturated fatty acids) = C14:0 + C15:0 + C16:0 + C17:0 + C18:0 + C + 20:0 + C22:0; ^3^ Total UFA (unsaturated fatty acids) = C15:1 + C16:1 + C17:1 + C18:1 + C18:2 + C18:3 + C20:4 + C20:5 + C22:6; ^4^ Total PUFA n-3 = C18:3 n-3 + C20:5 n-3 + C22:6 n-3; ^5^ Total PUFA n-6 = C18:2 n-6 + C20:2 n-6 + C20:3 n-6 + C20:4 n-6 + C22:2 n-6; ^6^ Total PUFA n-9 = C18:1 n9 + C20:1 n9 + C22:1 n-9; ^7^ Total PUFA = PUFA n-3 + PUFA n-6 + PUFA n-9; ^8^ Total MUFA (monounsaturated fatty acids) = C15:1 + C16:1 + C17:1 + C18:1; ^9^ SEM = standard error of mean; ^†^ The result of individual fatty acid was expressed as percentage from total fatty acid in each sample; ^a,b,c^ values with different superscripts within a row differ significantly at *p* < 0.05; * indicates that the value is significantly different at *p* < 0.05; ns indicates that the value is not significantly different at *p* < 0.05.

**Table 8:** Profit and loss analysis of buffaloes after being fed with different dietary treatments.

Breed	Murrah Cross		Swamp		SEM	*p*-Value		
Diets	Diet A	Diet B	Diet A	Diet B		Diet	Breed	Interaction
Revenue								
A. Meat sales (MYR)	2874.50 ^aY^	3754.31 ^bY^	2321.33 ^aZ^	2824.85 ^bZ^	257.80	*	*	*
B. Bone sales (MYR)	893.10	1266.72	646.88	708.50	120.80	*	*	*
C. Offal sales (MYR)	795.2	1094.10	669.55	843.15	77.14	*	*	*
D. Head sales (MYR)	70.00	70.00	70.00	70.00	-	-	-	-
E. Skin sales (MYR)	65.00	65.00	65.00	65.00	-	-	-	-
F. Tail sales (MYR)	10.00	10.00	10.00	10.00	-	-	-	-
G. Total revenue (MYR) (A + B + C + D + E + F)	4707.80 ^aY^	6260.13 ^bY^	3782.76 ^aZ^	4521.50 ^bZ^	901.60	*	*	*
Operating Expenses (2 years)								
Variable cost								
Cost of feeding (MYR/day)								
*Brachiaria* grass	0.71 ^a^	0.80 ^b^	0.66 ^a^	0.68 ^b^	0.03	*	ns	ns
Concentrate	1.46	1.43	1.36	1.23	0.04	ns	ns	ns
Bypass fat	-	0.75	-	0.65	0.03	ns	ns	ns
H. Total cost of average daily DMI (MYR/day/animal)	2.17 ^a^	2.98 ^b^	2.02 ^a^	2.56 ^b^	0.19	*	ns	ns
I. Total feed cost in 2 years (MYR/animal)	1580.44 ^a^	2171.62 ^b^	1477.74 ^a^	1867.33 ^b^	135.10	*	ns	ns
Fixed cost								
Management cost								
Deworming	0.50	0.50	0.50	0.50	-	-	-	-
ID tag	2.00	2.00	2.00	2.00	-	-	-	-
Fertilizer	156.00	156.00	156.00	156.00	-	-	-	-
Transportation	83.33	83.33	83.33	83.33	-	-	-	-
Labor cost	152.00	152.00	152.00	152.00	-	-	-	-
J. Total management cost (MYR/2 year/animal)	393.83	393.83	393.83	393.83	-	-	-	-
K. Pre- and post-inspection of slaughter services/animal (MYR)	5.00	5.00	5.00	5.00	-	-	-	-
L. Total cost (I + J + K)	1979.27 ^a^	2570.45 ^b^	1876.57 ^a^	2266.16 ^b^	135.10	*	ns	ns
Total net profit (MYR) (G-L)	2728.53 ^aY^	3689.68 ^bY^	1906.19 ^aZ^	2255.34 ^bZ^	335.04	*	*	*

Note: USD 1.00 = MYR 4.07 currency conversion 5 March 2021, MYR = Malaysian Ringgit. Estimations: income from fresh meat, MYR 31.03/kg; income from bone sales, MYR 26.00/kg, income from offal sales, MYR 35.00/kg, *Brachiaria* grass, MYR 0.23/kg dry matter; Concentrate mixture, MYR 1.11/kg; Bypass fat, MYR 3.82/kg. Diet A (control): 70% *Brachiaria decumbens* + 30% concentrate; Diet B (treatment): 70% *Brachiaria decumbens* + 26% concentrate + 4% bypass fat; SEM: standard error of means; ^a,b,Y,Z^: means with different superscript letters in the same column are significantly different at *p* < 0.05; * indicates that the value is significantly different at *p* < 0.05; ns indicates that the value is not significantly different at *p* < 0.05.

## Data Availability

Availability of data and equipment used and analyzed during this study is available from the correspondence author on reasonable request.

## Abbreviations

FA	Fatty acid
SS	*Supraspinatus* muscle
ST	*Semitendinosus* muscle
LTL	*Longissimus thoracis et lumborum*
SFA	Saturated fatty acid
UFA	Unsaturated fatty acid
MUFA	Monounsaturated fatty acid
PUFA	Polyunsaturated fatty acid
kg	Kilogram
MJ	Milli joule
GE	Gross energy
µg	Microgram
mg	Milligram
USA	United State of America
